# Computational thermodynamics and microstructure simulations to understand the role of grain boundary phase in Nd-Fe-B hard magnets

**DOI:** 10.1080/14686996.2020.1859339

**Published:** 2021-01-27

**Authors:** Toshiyuki Koyama, Yuhki Tsukada, Taichi Abe

**Affiliations:** a Department of Materials Design Innovation Engineering, Graduate School of Engineering, Nagoya University, Nagoya, Japan; b Computational Structural Materials Group, Research Center for Structural Materials, National Institute for Materials Science, Tsukuba, Japan

**Keywords:** Phase transformation, phase diagram, CALPHAD method, phase-field method, 407 CALPHAD, Phase field methods; 203 Magnetics, Spintronics, Superconductors

## Abstract

To control the coercivity of Nd hard magnets efficiently, the thermal stability of constituent phases and the microstructure changes observed in hard magnets during thermal processes should be understood. Recently, the CALPHAD method and phase-field method have been recognized as promising approaches to realize phase stability and microstructure developments in engineering materials. Thus, we applied these methods to understand the thermodynamic feature of the grain boundary phase and the microstructural developments in Nd-Fe-B hard magnets. The results are as follows. (1) The liquid phase is a promising phase for covering the Nd_2_Fe_14_B grains uniformly. (2) The metastable phase diagram of the Fe-Nd-B ternary system suggests that the tie line end of the liquid phase changes drastically depending on the average composition of Nd. (3) The Nd concentration in the grain boundary phase can reach 100 at% if the volume fraction of the grain boundary phase is constrained. (4) The effect of Cu addition to the Nd-Fe-B system on the microstructural morphology is reasonably modeled based on the phase-field method. (5) The morphology of the liquid phase can be controlled using phase separation in the liquid phase and the grain size of the Nd_2_Fe_14_B phase.

## Graphical Abstract

**Figure:**
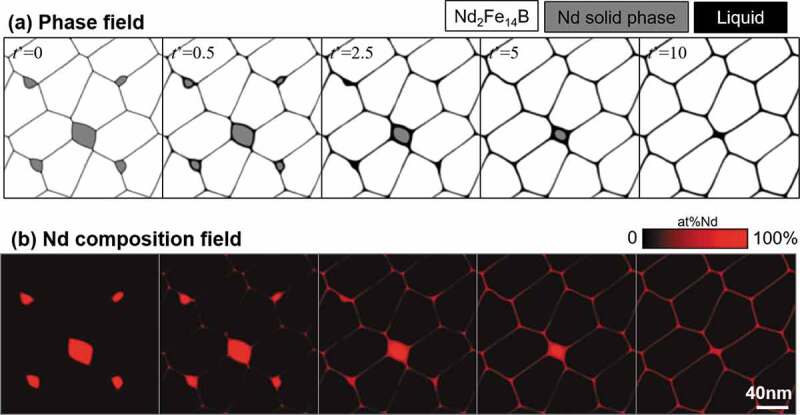

## 1. Introduction

Sintered Nd_2_Fe_14_B hard magnets have been improved continuously as the strongest permanent magnets since their discovery by Sagawa in 1984 [1]. It is well known that the coercivity of rare-earth hard magnets depends crucially on their internal microstructures. The high coercivity of Nd-Fe-B hard magnets is attributed to the thin grain boundary phase, which covers the Nd_2_Fe_14_B grains uniformly [2–4].

In this study, we focused on the grain boundary phase and investigated the thermodynamic stability and microstructural developments through computational thermodynamics and microstructure simulations using the CALPHAD method [5,6] and phase-field method [7–9], respectively. According to the literature on the grain boundary phase, the necessary conditions to achieve high coercivity are as follows [3,4]:

1. High volume fraction of the Nd_2_Fe_14_B phase (low volume fraction of grain boundary phase) is required to establish large magnetization.
2. The Nd_2_Fe_14_B grains are covered with a thin grain boundary phase uniformly.
3. The grain boundary phase is in a non-magnetic or a weak ferromagnetic state.
4. The grain size of the Nd_2_Fe_14_B phase is smaller. (It is estimated that the size is smaller than 1 μm to establish the coercivity $\mu_{0}H_{c}>2(T)$.) [4]

Other requirements, including aligning the magnetic easy axis of the Nd_2_Fe_14_B phase, controlling the grain shape of the Nd_2_Fe_14_B phase, and reducing defects such as a crack and a void, were excluded from this study because these are not directly related to the microstructure changes controlled by the grain boundary phase.

To control the internal microstructures of a hard magnet efficiently, we should understand the thermal stability of constituent phases and the microstructure changes observed in the magnets during their thermal processes. Given that the CALPHAD and phase-field methods have been recognized as effective approaches to realize the phase stability and microstructure developments in engineering materials recently [7–9], we applied these methods to understand the feature of grain boundary phase and the microstructure changes for controlling the coercivity of the magnets.

**Figure 1.**
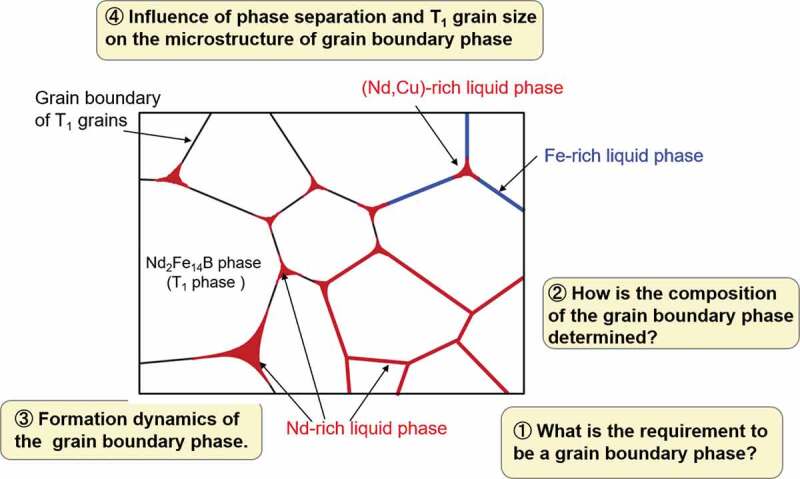
Schematic illustration of the microstructure in Nd magnet, where only a Nd_2_Fe_14_B phase (T_1_ phase) and a liquid phase (grain boundary phase) are considered. Items ①, ②, ③ and ④ are the four issues discussed in this study

The specific issues discussed in this paper are summarized as figure 1 that is a schematic illustration of the microstructure of Nd magnet, where only a Nd_2_Fe_14_B phase (T_1_ phase) and a liquid phase (grain boundary phase) are considered, and the other compound phases are out of scope in this study. To understand the role of the grain boundary phase in Nd-Fe-B hard magnets, we focused on the following four issues: ① What is the requirement to be a grain boundary phase? The reasons why the liquid phase is suitable for the grain boundary phase is explained in section 2. ② How is the composition of the grain boundary phase determined? The equilibrium and metastable approaches to estimate the composition of the grain boundary phase is discussed in Section 3. ③ The formation dynamics of the liquid phase at the grain boundary region of T_1_ grains. In section 4, the phase-field simulation is applied to the microstructure developments. ④ Discussion about the factors which affect the morphology of the grain boundary phase. The effects of phase separation in the liquid phase and grain size of the T_1_ phase on the morphology of the grain boundary phase are discussed in Section 5.

## 2. Physical requirement as a grain boundary phase

It is widely known that the characteristic microstructural morphology of the Nd_2_Fe_14_B phase, i.e. Nd_2_Fe_14_B grains are uniformly covered with a thin film of a grain boundary phase, is desirable to attain high coercivity. However, in the as-sintered state, there is no grain boundary phase in the microstructure of the magnet. The thin grain boundary phase is introduced during the optimization process, followed by sintering [3,4]. During the optimization process, a liquid phase appears at the grain boundary region in polycrystalline Nd_2_Fe_14_B grain microstructure because this thermal annealing is performed inside the two-phase region, Nd_2_Fe_14_B phase and liquid phase, on the phase diagram [3,4]. Furthermore, the high wetting ability of the liquid phase plays an important role in covering the Nd_2_Fe_14_B grains uniformly.

**Figure 2.**
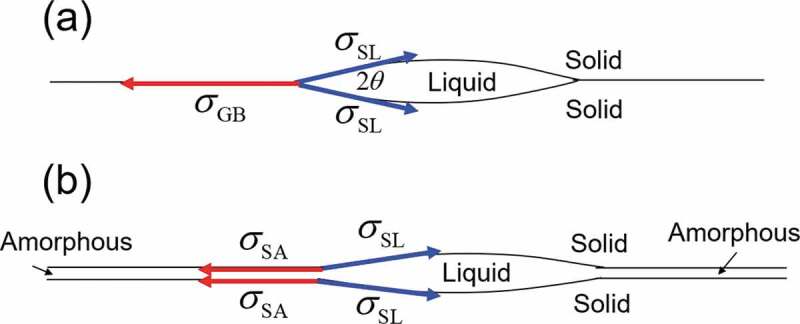
Schematic explaining the balance between interfacial energies

The wetting ability is estimated by the contact angle θ, defined in Young’s equation (equation (1)) [10], and the balance between interfacial energies is illustrated in figure 2, where $\sigma_{GB}$ and $\sigma_{SL}$ represent the grain boundary energy and solid/liquid interfacial energy, respectively. The relation among the variables θ, $\sigma_{GB}$, and $\sigma_{SL}$ is given as

(1) $$\sigma_{GB}=2\sigma_{SL}\cos(\theta ),\;\frac{\sigma_{GB}}{\sigma_{SL}}=2\cos(\theta )\leq 2.$$

As the experimental measurements of interfacial energy on common metals, such as Al, Cu, and Fe, provide $\sigma_{GB}/\sigma_{SL}≅1.5∼3.0$, the contact angle θ is small when a liquid phase exists at the grain boundary region [11,12]. This feature is clearly explained qualitatively in figure 2 (b). The grain boundary is often regarded as a thin-film amorphous phase in the phenomenological thermodynamic model, such as the grain-boundary-phase model [12,13] (refer to Section 3), where the Gibbs energy of the liquid phase is often used as that of the amorphous phase [12,13]. Therefore, the solid/amorphous interfacial energy $\sigma_{SA}$ can be approximated by $\sigma_{SL}$. This implies that the contact angle is θ≅0, then the liquid phase is an excellent phase for covering the Nd_2_Fe_14_B grains uniformly. Therefore, we reached the conclusion that the liquid phase is a promising phase as a grain boundary phase of Nd hard magnets.

In addition, achieving high thermodynamic stability of the Nd_2_Fe_14_B phase is important, because we can use the equilibrium condition between the Nd_2_Fe_14_B phase and a liquid phase to control the internal microstructural morphology produced during the thermal process. As the liquid phase is usually a stable phase at high temperature, if the Nd_2_Fe_14_B phase were a metastable phase or a low-stability stable phase, it would be difficult to determine the process conditions required to balance the Nd_2_Fe_14_B phase with a liquid phase, thermodynamically. In other words, almost all metastable phases will easily transform into stable phases when they contact with a liquid phase. Recently, the role of B in the Nd_2_Fe_14_B phase has been elucidated based on the first-principles calculations; that is, the addition of B to the Nd_2_Fe_14_ phase largely reduces the formation energy of Nd_2_Fe_14_B [14], then Nd_2_Fe_14_B phase is a stable phase which can coexist with a liquid phase at high temperature. Increasing the thermodynamic stability of the magnetic phase in hard magnets is the most important step when we utilize the liquid phase as a grain boundary phase.

Since the grain boundary phase should be in a non-magnetic or weak ferromagnetic state [3,4], calculating the composition of the grain boundary phase is beneficial because the magnetization is estimated approximately from the composition. This approach is explained in the next section.

## 3. Composition of the grain boundary phase

In this section, the composition of the grain boundary phase was calculated based on the conventional CALPHAD method and the phase-field method that is modified in accordance with the concept of the grain-boundary-phase model [12,13]. Although actual Nd hard magnets are multi-component alloys, we considered Fe-Nd-B ternary system in this section, for simplicity.

### 3.1 Metastable phase diagram

**Figure 3.**
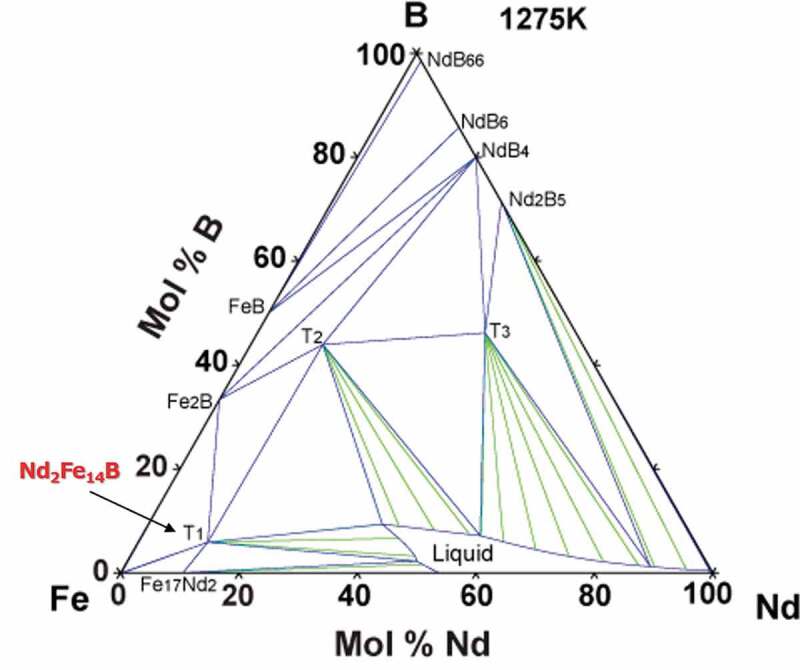
Equilibrium phase diagram of Fe-Nd-B ternary system at 1275 K, derived based on the CALPHAD method [15]

Figure 3 shows the calculated equilibrium phase diagram of the Fe-Nd-B ternary system at 1275 K [15], where T_1_ is the Nd_2_Fe_14_B phase and green lines indicate tie lines. Because of the high stability of the Nd_2_Fe_14_B phase, such phase is equilibrated with a liquid phase even at high temperature (i.e. 1275 K).

**Figure 4.**
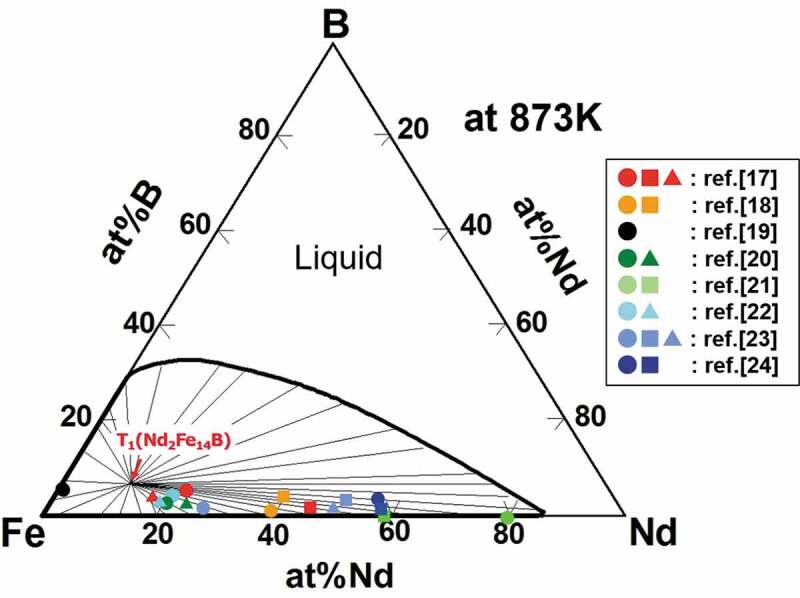
Metastable phase diagram of Fe-Nd-B ternary system at 873 K, which indicates the phase equilibria between T_1_ phase (Nd_2_Fe_14_B) and L phase (liquid) [16]

The practical thermal treatment to optimize the coercivity of the Nd magnet is annealing around 873 K, which promotes the formation of a thin liquid phase at the grain boundary region of T_1_ grains. It is important to understand the metastable phase equilibria between T_1_ phase and liquid phase at 873 K. Figure 4 shows the metastable phase diagram at 873 K calculated based on the conventional CALPHAD method, in which only two phases, T_1_ (Nd_2_Fe_14_B phase) and L (liquid phase), were considered [16]. The thin lines are tie lines connecting a T_1_ phase and a liquid phase. Note that there exits the metastable equilibrium between the T_1_ phase and the Nd-rich liquid phase at 873 K. We can easily know the composition of the metastable liquid phase from figure 4.

The colored symbols in the phase diagram indicate the compositions of the grain boundary phase [16], which have been obtained experimentally [17–24]. Although the positions of the symbols are scattered, all of them fall inside the (T_1_ + L) two-phase region, which will support the hypothesis that the grain boundary phase is related to the metastable liquid phase. Since the volume fraction of the T_1_ phase is dominant in an Nd magnet, the average alloy composition of Nd must be located beside the stoichiometric composition of the T_1_ phase in the (T_1_ + L) region. Therefore, the tie line end of the liquid phase changes drastically depending on the average concentration of Nd around the grain boundary region. The oxidation reaction of Nd is inevitable in rare-earth magnets [25]. Thus, a slight decrease in the Nd concentration in the local position of the microstructure due to the oxidation reaction results in a large concentration change of Nd in the metastable liquid phase, which may explain the scattering of the symbol positions in figure 4 [16].

### 3.2 Calculation of Nd composition in grain boundary phase

**Figure 5.**
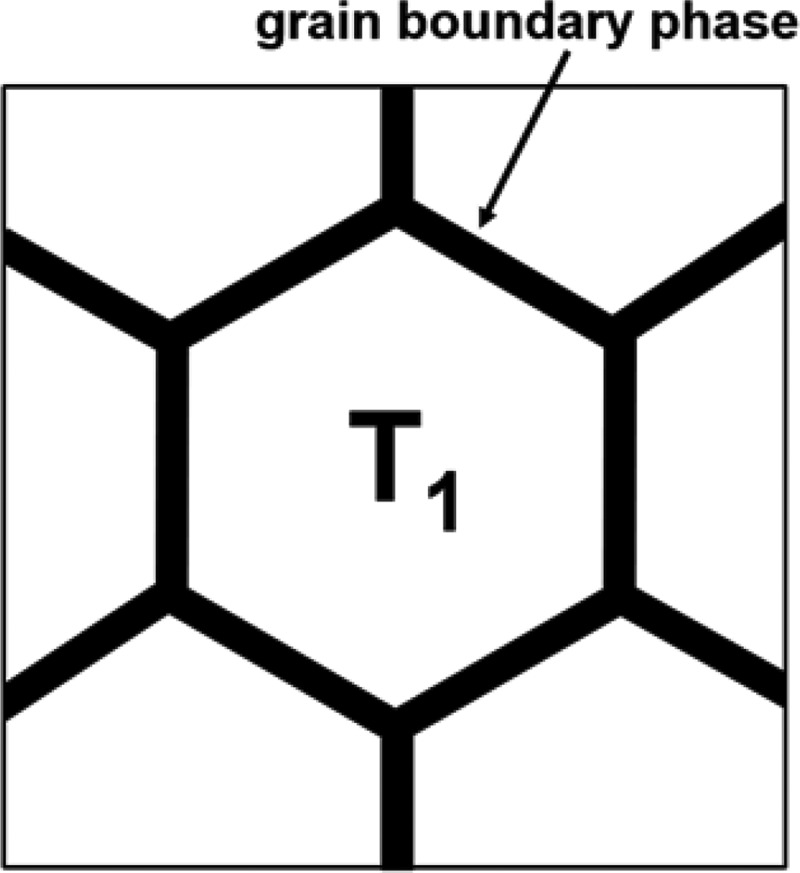
Schematic of the polycrystalline microstructure utilized to calculate the concentration in the grain boundary phase

Although the metastable phase diagram is useful as mentioned above, further modification is possible. The composition of the grain boundary phase was calculated by modifying the theory of the phase-field method based on the concept of the grain-boundary-phase model proposed by Hillert [12,13,16]. Figure 5 shows the schematic of the microstructure, which includes a T_1_ phase and a grain boundary phase. Experimental measurements elucidate that the grain boundary phase often takes an amorphous sate, with a width of ~3 nm [26]. In general, the volume fraction of a constituent phase is a variable in a conventional calculation of the phase diagram. However, as the width of the grain boundary phase is fixed, the volume fraction of the grain boundary phase is also fixed, as shown in figure 5. The phase equilibria under the condition of a constant volume fraction have been theoretically calculated based on the grain-boundary-phase model proposed by Hillert, where the parallel tangent construction to Gibbs energy curve is utilized to evaluate the grain boundary segregation [12,13]. The parallel tangent construction to the Gibbs energy curve is mathematically equivalent to that of the Gibbs energy minimization with the constraint of constant volume fraction. Hence, the composition of the grain boundary phase is calculated using the phase-field method, where the steady-state composition profile is simulated with the total free energy functional, which includes the penalty term that forces to maintain the volume fraction of the grain boundary phase a constant. Equation (2) shows the total free energy functional $G_{sys}$, and the last term in the integrand is the penalty term.

(2) $$\begin{array}{rl} & \;G_{sys}\\ & \;\;\;=\frac{1}{V}\int_{r}\left\{\begin{array}{c} \begin{array}{c} \;\;\;h\left(\sum_{i=1}^{N}\phi_{i}\right)G_{c}^{T_{1}}(c,T)+h(\phi_{0})G_{c}^{GBP}(c,T)\\ \;\;+\kappa (\nabla c{)}^{2} \end{array}\\ \begin{array}{c} \;\;\;+\sum_{i=1}^{N}\sum_{j=i+1}^{N}\left(-\frac{1}{2}\kappa_{ij}^{\;}\nabla \phi_{i}\cdot \nabla \phi_{j}+W_{ij}\phi_{i}\phi_{j}\right)\\ \;\;+\frac{1}{2}B{\left\{\frac{1}{V}\int_{r}h(\phi_{0})dr-f_{0}\right\}}^{2} \end{array} \end{array}\right\}dr, \end{array}$$

Here, $\phi_{0}(r,t)$ is the phase field of the grain boundary phase, which is a probability of finding the grain boundary phase in a microstructure at position r and time t. $f_{0}$ is the volume fraction of the grain boundary phase, $\phi_{i}(r,t)$(i=1∼N) are the phase fields of the T_1_ phase, and subscript i takes an integer number that distinguishes T_1_ grains with different crystal orientations from each other. $G_{c}^{T_{1}}(c,T)$ and $G_{c}^{GBP}(c,T)$ are the Gibbs energies of the T_1_ phase and the grain boundary phase, which are the functions of local composition c and temperature T, respectively. h(ϕ) is a monotone increasing function defined in the conventional phase-field method, and $\kappa ,\;\kappa_{ij}$ and $W_{ij}$ are the composition gradient energy coefficient, gradient energy coefficient of phase-field, and energy barrier forbidding the overlaps among T_1_ phases with different orientation numbers, respectively. *V* is the volume of material, and *B* is a positive constant that regulates the contribution of the penalty term. The composition of the grain boundary phase is determined from the steady-state composition profile obtained after the long-term phase-field simulation. The numerical values used in this simulation are $\kappa =\;\kappa_{ij}=4.0\times 10^{-15}[Jm^{2}/mol],\;W_{ij}=1.0\times 10^{4}(J/mol)$, and $B=1.0\times 10^{9}(J/mol)$, and the computation method is the same as the conventional multi-phase-field method; details are available in refs. [7–9]. Note that the microstructure morphology of figure 5 is fixed in this calculation because of the fixed volume fraction of grain boundary phase, and only the composition field is changed during phase-field simulation.

**Figure 6.**
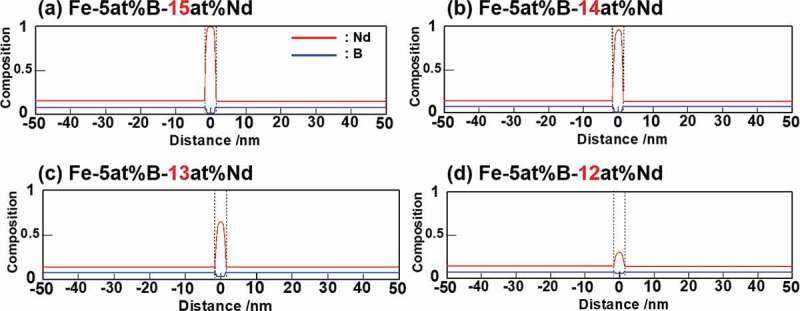
One-dimensional simulation of the steady-state composition profiles across the grain boundary region at 873 K [16]

Figure 6 shows one-dimensional simulation results of the steady-state composition profiles across the grain-boundary-phase region of Fe-B-Nd alloys at 873 K [16], where the B content was constant, 5 at%, and the Nd content was varied from 12 to 15 at% in the alloy composition. The position of a grain boundary phase is at the center of each profile; here, the width of the phase was fixed at 3 nm. Red and blue curves indicate each composition profile of Nd and B, respectively. The Gibbs energy of the liquid phase was used in place of that of the grain boundary phase, and the thermodynamic parameters with respect to Gibbs energies used in this study are summarized in Appendix A.

It is interesting to note that the Nd concentration in the grain boundary phase decreased drastically from 100 to 30 at% with a small change in the average Nd composition from 15 to 12 at%. The Nd composition in the grain boundary phase is significantly influenced even by a slight change in the Nd average composition. The equilibrium Nd composition in the grain boundary phase is ~80 at%, as per the calculation from the metastable phase diagram (figure 4). However, it reaches 100 at% in figure 6 if the volume fraction of the grain boundary phase is constrained. Meanwhile, the Fe concentration in the grain boundary phase is ～60 at% which closes to 83% of the alloy composition of Fe (Figure 6 (d)). This implies that the grain boundary phase is in a ferromagnetic state and will result in a decrease in the coercivity of the magnet [26]. Therefore, we reached the same conclusion in section 3.1 that the careful regulation with respect to the average composition of Nd around the grain boundary region of the T_1_ phase is key for developing high-performance Nd hard magnets.

The reason why the Nd composition of the grain boundary phase exceeded the Nd composition calculated in the metastable phase diagram can be easily understood if we imagine the parallel tangential construction to two Gibbs energy curves [13]. With reference to the common tangent contact point of the liquid phase (i.e. the equilibrium composition of liquid phase), the parallel tangent contact point must move to the outside of the two-phase region when the liquid phase is constrained to a small volume fraction relative to the equilibrium volume fraction, then the Nd composition in liquid phase becomes higher.

In addition, the calculation method utilized in this section is equivalent to the Lagrange multiplier method mathematically. When we ignore the gradient energy terms in eq. (2), the Hillert’s grain-boundary-phase model, the parallel tangential construction to Gibbs energy curve, is directly produced by minimizing eq. (2) with a constraint of fixed volume fraction of grain boundary phase. The advantage of the current calculation model is that we can consider the influence of the gradient energy. The relation between Hillert’s grain-boundary-phase model and our simulation method has already been discussed in ref. [16].

## 4. Microstructure simulations

In this section, two-dimensional simulations of the morphological microstructure changes of the as-sintered Nd hard magnet during isothermal aging, which includes the T_1_ phase, Nd solid phase, and liquid phase, were performed based on the phase-field method [27–29]; here, the liquid phase was regarded as the grain boundary phase. Therefore, we focused on the liquid phase microstructure developments at the grain boundary region of the T_1_ phase. Furthermore, the effect of Cu addition on the morphology of the microstructure is discussed [24].

Note that kinetic parameters, such as the diffusion coefficient and the mobility of the phase field, were considered constant in this study (refer to Appendix A). Therefore, the calculation results are mainly influenced by the driving force induced by the Gibbs energy and interfacial energy in the microstructure. The thermal process time was not discussed in this paper because our goal is to first understand the effect of driving forces.

### 4.1 Calculation conditions

**Figure 7.**
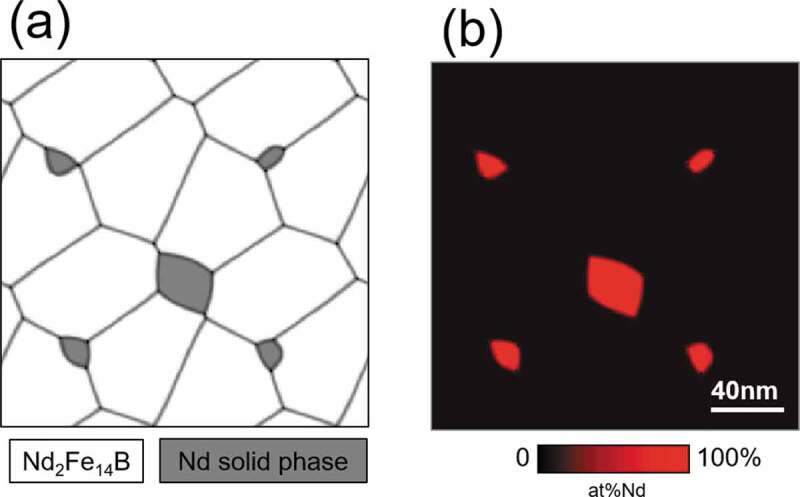
Initial microstructure utilized in the phase-field simulation, where the white and gray parts in (a) are the T_1_ phase and the Nd solid phase respectively, and the red color in (b) indicates the local Nd composition

Figure 7 shows an initial microstructure utilized in this simulation, where the white and gray parts in figure 7 (a) are the T_1_ phase and the Nd solid phase, respectively, and the gray lines represent the grain boundary of T_1_ grains. The morphology of polycrystalline microstructure of figure 7 was prepared by the conventional multi-phase-field method for simulating grain growth phenomena [8], and an isotropic microstructure morphology was assumed for simplicity, where we assigned Nd solid phase artificially. In this calculation, we considered a Fe-Nd-B ternary alloy and a Fe-Nd-B-Cu quaternary alloy. In the case of the Fe-Nd-B ternary alloy, the average composition is Fe-15.3 at% Nd-5 at% B, and the solute compositions in T_1_ phase and Nd solid phase of the initial microstructure (figure 7) are assigned to be Fe-12.0 at% Nd-5.2 at% B and pure Nd, respectively; here the composition in T_1_ phase was determined so as to reproduce the average composition of solute elements. On the other hand, in the case of Fe-Nd-B-Cu quaternary alloy, the average composition is Fe-15.3 at% Nd-5 at% B-0.20 at% Cu, and the solute compositions in T_1_ phase and Nd solid phase is assigned to be Fe-12.0 at% Nd-5.2 at% B-0.21 at% Cu and pure Nd, respectively. In the initial state, the composition at the grain boundary region of T_1_ grains was assumed to be the same as that of the T_1_ phase. Furthermore, the liquid phase nuclei were introduced at all triple junction points of T_1_ grains. The size of each nucleus is one squire pixel of the conventional finite difference method utilized in the phase-field simulation, and the value of the phase-field of each liquid nucleus is 1. The temporal evolution of liquid phase formation along the grain boundary region was simulated based on the phase-field method proposed in our previous work [30]. Detailed calculation methods are described in references [30,31]. The Gibbs energy parameters and material parameters utilized in this study are summarized in Appendix A.

### 4.2 Calculation results

**Figure 8.**
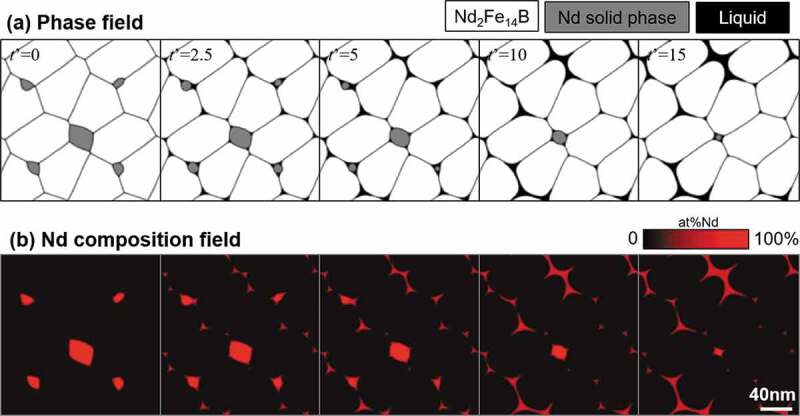
Phase-field simulation of the microstructure changes of Fe-15.3 at% Nd-5 at% B alloy with isothermal aging at 873 K

Figure 8 demonstrates the results of the two-dimensional simulation of the microstructural changes of Fe-15.3 at% Nd-5 at% B during isothermal aging at 873 K. Upper and lower figures show the phase and composition fields, respectively. The representation of the phase field is the same as that shown in figure 7, but the black region in the phase-field is the liquid phase, and the number indicated by *t*’ is dimensionless aging time. At the early stage, the Nd solid phase starts dissolving, and a liquid phase appears at the grain boundary region. With aging, the Nd solid phase gradually disappears, and the Nd-rich liquid phase penetrates along the grain boundary region. The liquid phase then agglutinates at the triple junction points of grain boundaries [30,32].

**Figure 9.**
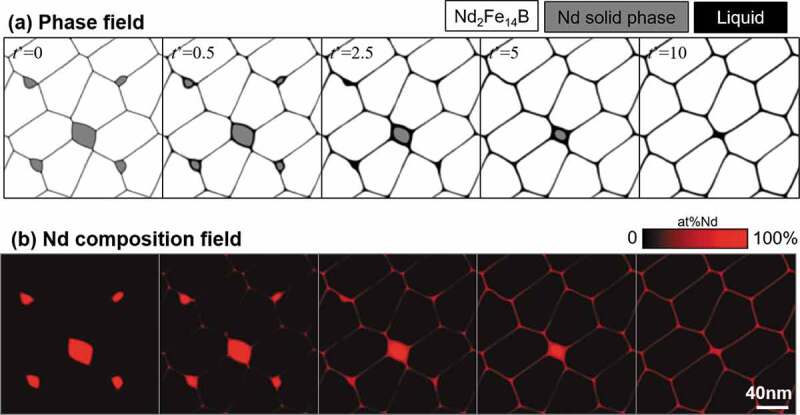
Phase-field simulation of the microstructure changes of Fe-15.3 at% Nd-5 at% B-0.2 at% Cu alloy with isothermal aging at 873 K

Figure 9 shows the simulation result of the Fe-15.3 at% Nd-5 at% B-0.2 at% Cu alloy under the same conditions as those presented in figure 8. It is interesting to note that the characteristic microstructural morphology, i.e. uniform coverage of the T_1_ grains with the liquid phase, was formed. It has been elucidated experimentally that the Cu addition lowers the melting point of the liquid phase because an eutectic reaction exists in the Cu-Nd binary phase diagram [33]. By increasing the stability of the liquid phase by Cu addition, the melting point of the liquid phase is decreased, which usually lowers the interfacial energy $\sigma_{SL}$ in figure 2 and the contact angle θ also decreases. Then, the characteristic morphology (i.e. uniform coating of T_1_ grains by the liquid phase) is stabilized relatively.

According to the calculation theory of the phase-field method, interfacial energy is defined as a sum of the gradient energy and the Gibbs energy at the interface region. The gradient energy coefficient is the same value in the simulations of figures 8 and 9. Therefore, according to the different behavior of the liquid phase between figures 8 and 9, we can recognize that the Gibbs energy at the interfacial region is influenced by the Cu addition. As mentioned above, it has been known that the Cu addition lowers the melting point of the liquid phase, which provides a decrease of Gibbs energy of the liquid phase, then the Gibbs energy at the interfacial region will be also decreased. As the result, the interfacial energy was lowered. It should be emphasized that this effect is automatically included in the phase-field simulation. We can conclude that the phase-field simulation is an effective and useful approach for understanding the developments of microstructures observed in Nd hard magnets involving the grain boundary phase.

## 5. Discussion

In this paper, since we are interested in the morphological stability of the liquid-phase microstructure, the effects of the phase separation in the liquid phase and the grain size of the T_1_ phase on the morphological changes of the liquid-phase microstructure are discussed in this section by using the phase-field simulation. To understand the microstructure changes concisely, we employed simple calculation conditions here, i.e. only two phases, liquid phase and T_1_ phase, were considered and the Nd solid phase was excluded in this section.

### 5.1 Phase separation in the liquid phase

**Figure 10.**
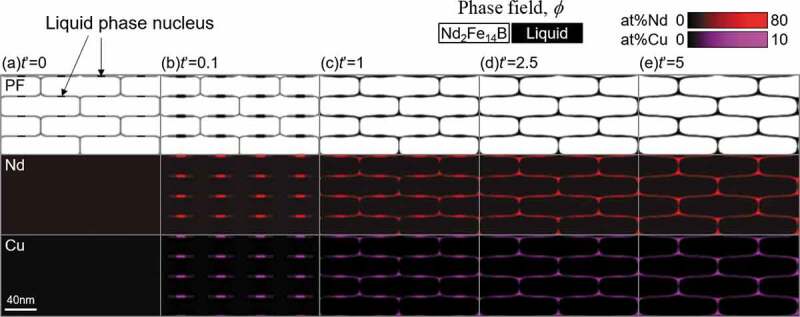
Phase-field simulation of the liquid phase formation in Fe-16 at% Nd-5 at% B-0.5 at% Cu alloy with isothermal aging at 873 K. The liquid phase nucleus was initially introduced at the horizontal grain boundary region

Figure 10 shows the phase-field simulation of the liquid phase formation at the grain boundary region in Fe-16 at% Nd-5 at% B-0.5 at% Cu alloy during isothermal aging at 873 K. The upper, middle, and lower layers in figure 10 are the phase-field, Nd composition field, and Cu composition field, respectively. The local composition is indicated by the brightness of the color (see the color scale in figure 10). The initial microstructure is represented as figure 10 (a), in which the composition field is uniform and the liquid phase nuclei are introduced (see the positions indicated by arrows). Inside the liquid phase nuclei, the value of the liquid phase-field is 1. As for the polycrystalline microstructure morphology of T_1_ grains, we referred to the microstructures observed in the anisotropic Nd-Fe-B hot-deformed magnets [34].

**Figure 11.**
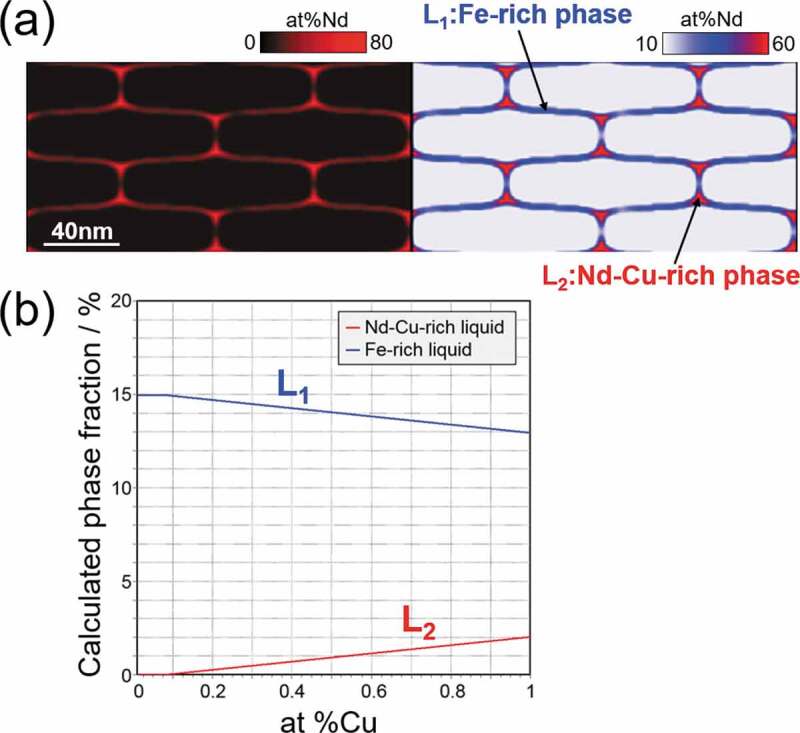
The local Nd concentration in the calculated microstructure (e) in Fig. 10, and the phase fraction of each liquid phase, L_1_ and L_2_, depending on the Cu alloy composition calculated based on the CALPHAD method

It is recognized that the components, Nd and Cu, are mainly partitioned to the liquid phase with aging. When we focus on the local compositions of Nd and Cu in figure 10 (e), the brightness of the red color is different between the triple junction region and the grain boundary region of the T_1_ polycrystalline microstructure. This is because of the phase separation in the liquid phase, and the effect of phase separation on the morphological stability of the liquid phase, which covers the T_1_ grains, was analyzed in detail. Figure 11 (a) shows the same microstructure (Nd composition field) as that shown in figure 10 (e), and the right-hand side image shows the different representation of the composition field. The blue and red parts indicate the Fe-rich liquid phase (L_1_) and Nd-Cu-rich liquid phase (L_2_), respectively. As the phase separation of the liquid phase has been reported in the thermodynamic assessment of the Fe-Cu-Nd phase diagram [35], the liquid phase separation observed in figure 11 (a) is due to the Cu addition. Figure 11 (b) shows the phase fraction of each liquid phase, L_1_ and L_2_, which is dependent on the Cu alloy composition, calculated using the CALPHAD method. The phase fraction of the L_2_ phase gradually increases with increasing Cu composition, whereas the phase fraction of the L_1_ phase gradually decreases, then the total phase fraction of the liquid phases is not greatly affected by the Cu composition. For increasing the total phase fraction of the liquid phases, it requires the simultaneous increase both of Nd and Cu contents in the alloy composition.

In the microstructure shown in figure 11 (a), the L_2_ phase is mainly detected at the triple junction region of T_1_ grains, whereas the L_1_ phase is observed at the grain boundary region between T_1_ grains. It should be emphasized that this layout of each liquid phase may slow down the coarsening of each liquid phase. If we imagine the coarsening process of the L_1_ phase, the L_1_ phase should move over the L_2_ phase; in other words, the movements of the L_1_ and L_2_ phases will interfere with one another during coarsening. The phase separation of the liquid phase can contribute to stabilize the characteristic microstructural morphology (uniform coating of the T_1_ grains by the liquid phase) temporally.

**Figure 12.**
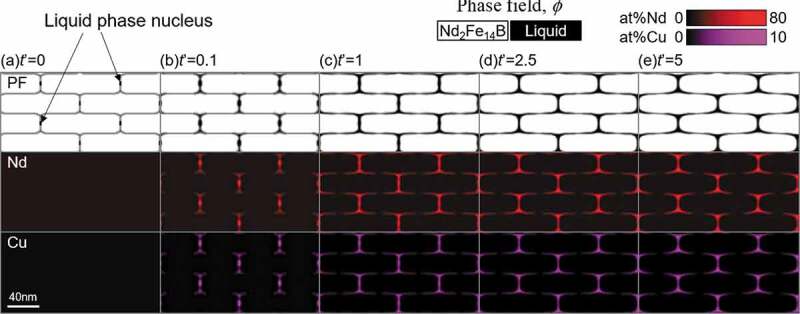
Phase-field simulation of the liquid phase formation in Fe-16 at% Nd-5 at% B-0.5 at% Cu alloy with isothermal aging at 873 K. The liquid phase nucleus was initially introduced at the vertical grain boundary region

In addition, to clarify the effect of the position of liquid phase nuclei, the simulation in which we changed the position of the nuclei was performed as shown in figure 12 (see the arrows in figure 12 (a)). The other calculation conditions are not changed from that of figure 10. It is interesting that the Nd-Cu-rich liquid phase (L_2_ phase) mainly settles at the triple junction points of the grain boundary region of T_1_ grains as in the case of figure 10. However, the width of the L_2_ phase becomes relatively larger compared with the case of figure 10, which is because the liquid phase state was maintained for a long time at this position in figure 12.

Although the reason why the L_2_ phase preferentially occupies the triple junction points of T_1_ grains is currently under consideration, one possible explanation may be as follows: Since the Gibbs energy of L_2_ phase is lower than that of L_1_ phase because Nd and Cu compositions in L_2_ phase are higher than those of L_1_ phase. As the energy at the triple junction points of the grain boundary is high, if the L_2_ phase occupies these regions, the interfacial energy will be lowered relative to the case of the L_1_ phase. Because the phase-field method automatically finds the low energy state, it can be understood that the preferential occupation of the L_2_ phase at the triple junction points of T_1_ grains is calculated.

### 5.2 Effect of the grain size of the T_1_ phase

**Figure 13.**
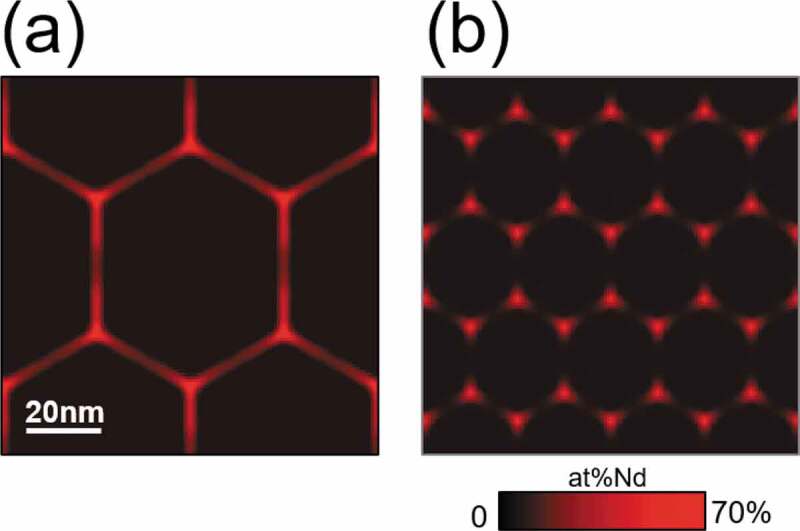
Phase-field simulation results of the liquid-phase microstructures in Fe-15 at% Nd-5 at% B-0.2 at% Cu alloy with isothermal aging at 873 K, where the initial microstructure is changed: the grain size of the T_1_ phase in (a) is larger than that in (b)

Figure 13 shows the phase-field simulation of the liquid-phase microstructure formation in Fe-15 at% Nd-5 at% B-0.2 at% Cu alloy during isothermal aging at 873 K, where the initial microstructure of T_1_ grains was changed, and the simple hexagonal polycrystalline microstructure is employed for simplicity. The grain size of the T_1_ phase in (a) is larger than that in (b). Other calculation conditions are the same as those shown in figure 10.

Almost all of the T_1_ grains in figure 13(b) are not covered by the liquid phase. When the average size of the T_1_ grains is small, the total grain boundary area in the material becomes large. Therefore, a large amount of the liquid phase is required to cover the T_1_ grains uniformly. Note that the suitable solute composition of alloys and optimum thermal process conditions, which results in the ideal microstructure (T_1_ grains are uniformly covered with a grain boundary phase), depending on the grain size and shape of the T_1_ phase. The small grain size of the T_1_ phase is attractive to improve the coercivity of Nd hard magnets [3,4]. The optimization using microstructure simulations is an effective strategy for determining the optimal microstructures and process conditions, such as alloy compositions and grain size of the T_1_ phase.

## 6. Conclusions

In this study, we focused on the grain boundary phase and investigated the thermodynamic stability and the microstructure developments by using computational thermodynamics and microstructure simulations, which were performed using the CALPHAD method and phase-field method, respectively. The conclusions obtained are as follows:

1. The liquid phase is a promising phase for covering the Nd_2_Fe_14_B grains uniformly, which is helpful for attaining high coercivity. It is important to recognize that this concept is supported by the high thermodynamic stability of the T_1_ phase.
2. The metastable phase diagram of the Fe-Nd-B ternary system suggests that the tie line end of the liquid phase changes drastically depending on the average Nd composition. The oxidation reaction of Nd is inevitable in rare-earth magnets; thus, a slight decrease in the Nd concentration in the local position of the microstructure results in a large concentration change of Nd in the metastable liquid phase.
3. Nd concentration in the grain boundary phase can reach 100 at% if the volume fraction of the grain boundary phase is constrained, even if the maximum Nd concentration determined from the metastable phase diagram is less than 100 at%. This provides flexibility to control the concentration of the grain boundary phase from the viewpoint of microstructures.
4. Phase-field simulations are useful for understanding the morphological changes in the microstructures, including the grain boundary phase. In particular, the effect of Cu addition on the microstructural morphology is reasonably modeled based on the phase-field method.
5. The morphology of the liquid phase can be controlled using phase separation in the liquid phase and the grain size of the T_1_ phase.

## Appendix A

The thermodynamic Gibbs energy parameters utilized in this study are summarized as follows in Table A1.

**Table A1.** Thermodynamic Gibbs energy parameters utilized in this study

Thermodynamic Gibbs energy parameters/$J\cdot mol^{-1}$		Temperature, *T*/K
GHSERFE	$\begin{array}{rl} & \;\;\;\;\;1225.7+124.134T-23.5143T\ln(T)\\ & -0.00439752T^{2}-5.89269\times 10^{-8}T^{3}+77358T^{-1} \end{array}$	$\left[298.15\leq T<1811\right]$ [36]
GHSERND	$\begin{array}{rl} & -8402.93+111.10239T-27.0858T\ln(T)\\ & \;+5.56125\times 10^{-4}T^{2}-2.6923\times 10^{-6}T^{3}+34887T^{-1}\; \end{array}$	$\left[298.15\leq T<900\right]$ [36]
GHSERBB	$\begin{array}{rl} & -7735.284+107.111864T-15.6641T\ln(T)\\ & \;-0.006864515T^{2}+6.18878\times 10^{-7}T^{3}+370843T^{-1} \end{array}$	$\left[298.15\leq T<1100\right]$ [36]
GHSERCU	$\begin{array}{rl} & -7770.458+130.485235T-24.112392T\ln(T)\\ & -2.65684\times 10^{-3}T^{2}+0.129223\times 10^{-6}T^{3}+52478T^{-1} \end{array}$	$\left[298.15\leq T<1357.77\right]$ [36]
${\;}^{0}G_{Fe}^{L}$	$\begin{array}{rl} & 13265.87+117.57557T-23.5143T\ln(T)\\ & \;-4.39752\times 10^{-3}T^{2}-0.058927\times 10^{-6}T^{3}\\ & \;+77359T^{-1}-367.516\times 10^{-23}T^{7} \end{array}$	$\left[298.15\leq T<1811\right]$ [36]
${\;}^{0}G_{Nd}^{L}$	$\begin{array}{rl} & 5350.01-86.593963T+5.357301T\ln(T)\;\\ & \;-0.046955463T^{2}+6.860782\times 10^{-6}T^{3}-374380T^{-1} \end{array}$	$\left[298.15\leq T<1128\right]$ [36]
${\;}^{0}G_{B}^{L}$	$\begin{array}{rl} & 41119.703+82.101722T-14.9827763T\ln(T)\\ & \;-0.007095669T^{2}+5.07347\times 10^{-7}T^{3}+335484T^{-1}\; \end{array}$	$\left[500<T<2348\right]$ [36]
${\;}^{0}G_{Cu}^{L}$	$\begin{array}{rl} & 5194.277+120.973331T-24.112392T\ln(T)-2.65684\times 10^{-3}T^{2}\\ & +0.129223\times 10^{-6}T^{3}+52478T^{-1}-584.9\times 10^{-23}T^{7} \end{array}$	$\left[298.15\leq T<1357.77\right]$ [36]
${\;}^{0}L_{\left(Fe,Nd\right)}^{L}$	3221	[37]
${\;}^{1}L_{\left(Fe,Nd\right)}^{L}$	6216	[37]
${\;}^{2}L_{\left(Fe,Nd\right)}^{L}$	4826	[37]
${\;}^{0}L_{\left(B,Fe\right)}^{L}$	−133438+33.95T	[37]
${\;}^{1}L_{\left(B,Fe\right)}^{L}$	7771	[37]
${\;}^{2}L_{\left(B,Fe\right)}^{L}$	29739	[37]
${\;}^{0}L_{\left(B,Nd\right)}^{L}$	−123735+42.87T	[37]
${\;}^{1}L_{\left(B,Nd\right)}^{L}$	−41869	[37]
${\;}^{0}L_{\left(B,Cu\right)}^{L}$	−3156.2	[38]
${\;}^{1}L_{\left(B,Cu\right)}^{L}$	14390.26−9.98T	[38]
${\;}^{2}L_{\left(B,Cu\right)}^{L}$	42620−20T	[38]
${\;}^{0}L_{\left(Cu,Fe\right)}^{L}$	73316.72−142.79T+15.82Tln(T)	[39]
${\;}^{1}L_{\left(Cu,Fe\right)}^{L}$	9100.15−5.94T	[39]
${\;}^{2}L_{\left(Cu,Fe\right)}^{L}$	2428.96	[39]
${\;}^{3}L_{\left(Cu,Fe\right)}^{L}$	−233.62	[39]
${\;}^{0}L_{\left(Cu,Nd\right)}^{L}$	−55583.6+5.8955T−2.4901Tln(T)	[40]
${\;}^{1}L_{\left(Cu,Nd\right)}^{L}$	−27795.1+3.1830T−1.2911Tln(T)	[40]
${\;}^{2}L_{\left(Cu,Nd\right)}^{L}$	−3806	[40]
${\;}^{0}G^{T_{1}}$	$\frac{2}{17}GHSERND+\frac{14}{17}GHSERFE+\frac{1}{17}GHSERBB-11312+2.813T$	[37]
${\;}^{0}G^{\alpha }$	GHSERND	[37]
$A_{Nd}^{T_{1}}$	$5.0\times 10^{6}$	[This work]
$A_{B}^{T_{1}}$	$1.0\times 10^{7}$	[This work]
$A_{Cu}^{T_{1}}$	$5.0\times 10^{6}$	[This work]
$A_{Nd}^{\alpha }$	$5.0\times 10^{6}$	[This work]
$A_{Cu}^{\alpha }$	$5.0\times 10^{6}$	[This work]
$A_{B}^{\alpha }$	$5.0\times 10^{6}$	[This work]

The notations comply with the conventional CALPHAD format [6].

The Gibbs energy curve of the T_1_ phase is assumed to be represented by a quadric surface with respect to compositions as follows:

(A1) $$G^{T_{1}}(c_{Nd},c_{B},c_{Cu},T)={\;}^{0}G^{T_{1}}(T)+A_{Nd}^{T_{1}}{\left(c_{Nd}-\frac{2}{17}\right)}^{2}+A_{B}^{T_{1}}{\left(c_{B}-\frac{1}{17}\right)}^{2}+A_{Cu}^{T_{1}}{\left(c_{Cu}-10^{-4}\right)}^{2}.$$

As Cu atoms hardly dissolve into the T_1_ phase, the solubility limit of Cu was set as 10^−4^ in eq. (A1). Meanwhile, we assumed the Gibbs energy of the Nd solid phase (here, the phase name is denoted as α), as expressed in eq. (A2)

(A2) $$G^{Nd\;solid\;phase}={\;}^{0}G^{\alpha }(T)+A_{Nd}^{\alpha }{\left(c_{Nd}-0.99\right)}^{2}+A_{B}^{\alpha }{\left(c_{B}-0.005\right)}^{2}+A_{Cu}^{\alpha }{\left(c_{B}-0.005\right)}^{2}.$$

Since we assumed the Nd solid phase to be a pure Nd, the first term on the right-hand side of eq. (A2) is the Gibbs energy of pure Nd crystalline phase with a double-hexagonal-close-packed structure.

The material parameters and calculation conditions for phase-field simulations are summarized in Table A2.

**Table A2.** Numerical values used for the phase-field simulations

Unit grid size for finite difference method, Δx/nm	0.8	
Interfacial thickness, *δ*/nm	5Δx	
Permeability parameter, *P*	1.0	[30]
Interfacial mobility of phase-field*, $M_{ij}^{\;}$	5.0*	
Diffusion coefficient of solute element, $D_{0}$	1000*	
Interfacial energy density/$J\cdot m^{-2}$		
$\sigma_{T_{1}/T_{1}},\;\sigma_{T_{1}/\alpha }$	1.5	[41]
$\sigma_{T_{1}/L},\;\;\sigma_{\alpha /L}$	0.76	[This work]

* Dimensionless value.

All the diffusion constants for solute elements in solid and liquid phases are assumed to be the same, and all the phase-field mobilities, which are the relaxation constants governing the local change rate of phase-field, are considered constant for simplicity. Although microstructural developments depend on these kinetic material parameters, we ignored these effects in this calculation because we needed to understand first the effect of the energetic driving force on the microstructure changes. Therefore, we used the Gibbs energy parameters which have been assessed in the CALPHAD method. The grain boundary energy of Fe polycrystal was employed for $\sigma_{T_{1}/T_{1}}$ [41], and we assumed $\sigma_{T_{1}/\alpha }=\sigma_{T_{1}/T_{1}}$. The $\sigma_{T_{1}/L}=\sigma_{\alpha /L}=0.76$ was determined to hold the contact angle $\theta =10^{\circ }$ in equation (1).
